# Repair Bond Strength to Hybrid CAD/CAM Materials after Silane Heat Treatment with Laser

**DOI:** 10.3290/j.jad.b3956401

**Published:** 2023-03-15

**Authors:** Ceren Deger, Burcu Oglakci, Zumrut Ceren Ozduman, Evrim Eliguzeloglu Dalkilic

**Affiliations:** a Assistant Professor, Bezmialem Vakif University, Faculty of Dentistry, Department of Restorative Dentistry, Istanbul, Turkey. Idea, experimental design, performed the experiments, wrote the manuscript.; b Associate Professor, Bezmialem Vakif University, Faculty of Dentistry, Department of Restorative Dentistry, Istanbul, Turkey. Hypothesis, performed the experiments, wrote the manuscript.; c Assistant Professor, Bezmialem Vakif University, Faculty of Dentistry, Department of Restorative Dentistry, Istanbul, Turkey. Performed the experiments, contributed substantially to discussion, proofread the manuscript.; d Professor, Bezmialem Vakif University, Faculty of Dentistry, Department of Restorative Dentistry, Istanbul, Turkey. Performed the experiments, contributed substantially to discussion, proofread the manuscript.

**Keywords:** shear bond strength, CAD/CAM, silane heat treatment, Er:YAG laser.

## Abstract

**Purpose:**

This study investigated the effect of different surface treatments and the effect of silane heat treatment with laser on the shear bond strength (SBS) of a nanoceramic composite to repaired hybrid CAD/CAM blocks.

**Materials and Methods:**

60 hybrid CAD/CAM specimens (Cerasmart, GC) were prepared and randomly divided into six groups according to the different surface treatments (n = 10): group ER: Er:YAG laser+silane (Monobond Plus, Ivoclar Vivadent); group ER+SHT: Er:YAG laser+silane heat treatment; group B: bur+silane; group B+SHT: bur+silane heat treatment; group HF: hydrofluoric acid+silane; group HF+SHT: hydrofluoric acid+silane heat treatment. Afterwards, a universal adhesive (Universal Bond Quick, Kuraray) was applied, and nanoceramic resin composite (Zenit, President) cylinders were bonded to the Cerasmart specimens. They were thermocycled for 10,000 cycles (5–55°C) and subjected to SBS testing using a universal testing machine. Failure modes were examined with a stereomicroscope (15X). Scanning electron microscopy (SEM) was used to evaluate the surface topography (n = 2). The data were statistically analyzed using the Mann-Whitney U-test and the Kruskal-Wallis test (p < 0.05).

**Results:**

Regarding the surface treatments, group ER showed significantly lower SBS than groups B and HF (p < 0.05). Regarding the presence of silane heat treatment by laser, groups ER+SHT and B+SHT showed significantly lower SBS than group HF+SHT(p < 0.05). In addition, group B+SHT showed significantly lower SBS than did group B (p < 0.05).

**Conclusion:**

Er:YAG laser treatment for repairing hybrid CAD/CAM blocks was not as effective as bur roughening or hydrofluoric acid etching. Silane heated by Er:YAG laser was incapable of significantly increasing the bond strength to repaired hybrid CAD/CAM blocks.

Computer-aided design/computer-aided manufacturing (CAD/CAM) technologies have become an effective alternative to conventional dental restorative methods with the use of advanced design software and advanced biomaterials. Ceramic CAD/CAM blocks have many advantages, such as high fracture resistance, low material wear, and color stability.^47,29^ However, some studies have reported failures due to the rigid nature of ceramic materials^35^ and potential abrasive effects on opposing teeth.^41^ In addition to the advantages provided by ceramic materials, manufacturers have developed hybrid blocks with the same advantages as dental resin composites, including low abrasiveness and an elastic modulus similar to that of human dentin.^12^ Cerasmart (GC; Tokyo, Japan), a nanoparticle-filled resin, is one of these materials and has a 71% filler ratio. The fact that restorations made of hybrid materials are produced in a single appointment without the need for sintering or glazing is another advantage. However, regardless of the material, the clinical life span of dental restorations may be limited due to fractures as a result of parafunctional habits and internal stress.^36^

Repairing CAD/CAM restorations can be beneficial, since the procedure is less invasive and decreases the risk of damaging healthy dental tissues, in addition to being cost-effective. Moreover, repairing a dental restoration may reduce chairside time and the cost of treatment.^8,25^ Roughening with diamond burs, air abrasion, acid etching, or laser irriadiation^1^ can be performed as surface treatments to ensure a durable bond between repair material and CAD/CAM blocks. Nevertheless, there is limited data on the efficacy of universal dental adhesives in combination with various surface treatments of resin nanoceramic and hybrid ceramic CAD/CAM block materials.^3,40^

It is reported that erbium:yitriium aluminum garnet (Er:YAG) laser irradiation increases the bond strength of dental restorative materials by creating microretentive surfaces.^39^ Additionally, the use of a silane coupling agent is recommended to enhance the wettability of the surface to create effective adhesion. A silane heating procedure can improve the silane condensation reaction with the help of durable covalent bonds.^26^ To the authors’ knowledge, the literature contains only limited data regarding the effect of laser applications for silane heat treatment (SHT) on the adhesion of CAD/CAM hybrid materials.^7,24^

Further research is required, as previous studies used various laser parameters and reported conflicting results.^24^ The results of laser-heated silane procedures are also unclear. Thus, the purpose of this study was to investigate the effect of different surface treatments and laser heat treatment of silane on the shear bond strength (SBS) of a nanoceramic composite to repaired CAD/CAM hybrid blocks.

The null hypotheses of this study were: 1. Different surface treatments would not affect the shear bond strength of the nanoceramic composite to repaired CAD/CAM hybrid blocks. 2. Laser heat treatment would not affect the shear bond strength of the nanoceramic composite to repaired CAD/CAM hybrid blocks.

## Materials and Methods

### Specimen Size Calculation

A power analysis was performed to establish specimen size according to a previous study.^24^ In this study, for each group, a minimum of 10 specimens was required to gain a medium effect size (d = 0.50) with 90% power and a 5% type-1 error rate.

### Specimen Preparation and Restorative Procedures

**Fig 1:**
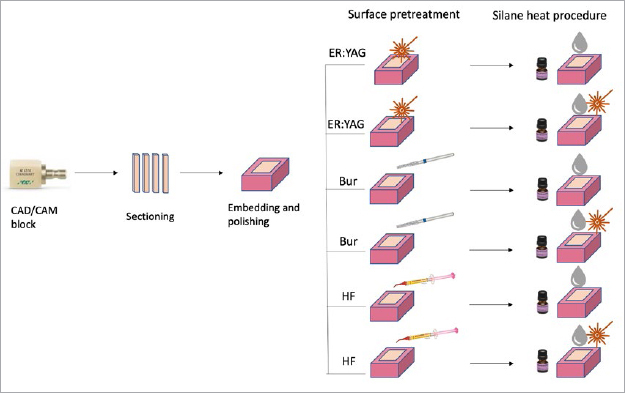
Schematic description of specimen preparation.

The chemical compositions and brands of the restorative materials used are summarized in Table 1. Sixty hybrid CAD/CAM blocks (Cerasmart, GC; Tokyo, Japan) were cut with a diamond-coated disk (Dimos, Ø125, Metkon; Bursa, Turkey) into bars with dimensions of 5 x 10 x 10 mm^3^ and embedded in acrylic molds. The surfaces were prepared by polishing with silicon carbide papers (400, 600, 800, and 1000 grit). They were then randomly divided into six groups for different surface treatments (n = 12). Ten specimens from each group were evaluated with shear bond strength testing, and two specimens from each group were examined using SEM (Hitachi S-4800 FEG Scanning Electron Microscope, Hitachi; Tokyo, Japan). Specimen preparation is illustrated schematically in Fig 1.

**Table 1:** The restorative materials used, their compositions, and brand numbers

Brand names	Manufacturer	Composition	Lot number
Cerasmart	GC; Tokyo, Japan	Bis-MEPP, UDMA, DMA with 71 wt% silica and barium glass nanoparticles	2007281
Monobond Plus	Ivoclar Vivadent; Schaan, Liechtenstein	MPTMS, 10-MDP, disulfide dimethacrylate, ethanol	Z00WXC
Clearfil Universal Bond Quick	Kuraray Noritake; Tokyo, Japan	Bis-GMA, HEMA, ethanol, 10-MDP. hydrophilic aliphatic dimethacrylate, colloidal silica, CQ, silane coupling agent, accelerators, initiators, water	7B0219
Zenit	President; Munich, Germany	Glass filler, pyrogenic silica, agglomerated nanoparticles, diurethane dimethacrylate, butanediol dimethacrylate, isopropylide- bis [2(3)-hydroxy-3(2)-(4-phenoxy) propyl] bis-methacrylate	2019009884

Bis-GMA: bisphenol A glycidyl methacrylate; bis-MPEPP: bis-methacryloxyethoxy phenyl propane; DMA: dodecyl dimethacrylate; HEMA: 2 hydroxyethyl methacrylate; MDP: methacryloyloxydecyl dihydrogen phosphate; MPTMS: γ-methacryloxypropyl trimethoxysilane; UDMA: urethane dimethacrylate; TEG-DMA, triethylene glycol dimethacrylate; wt%, weight percentage.

- Group ER (Er:YAG laser etching + silane): An Er:YAG laser (AT Fidelis, Fotona; Ljubljana, Slovenia) was applied to the hybrid block surface for 30 s in non-contact mode (20 Hz, long pulse, 5 W and 250 mJ).^15^ Silane (Monobond Plus, Ivoclar Vivadent, Liechtenstein) was then applied and allowed to dry for 60 s.
- Group ER+SHT (Er:YAG laser etching + silane heat treatment): In addition to surface pretreatment procedures in group ER, the silane was heated with Er:YAG laser irradiation according to the parameters in group ER.
- Group B (bur roughening + silane): The hybrid block surfaces were abraded with a coarse-fissure diamond bur (green band, 125–150 µm) (Le Blond A&M Instruments; Alpharetta, GA, USA), and then silane was applied as described above.
- Group B+SHT (bur roughening+ silane heat treatment): After the hybrid block surfaces were abraded with a coarse-fissure bur, silane was applied and then heated by Er:YAG laser irradiation according to the parameters in group ER.
- Group HF (hydrofluoric acid etching + silane): The hybrid blocks were washed and dried after 90 s of hydrofluoric acid etching (Ultradent Porcelain Etch, Ultradent; South Jordan, UT, USA), then the silane procedure was performed as described above.
- Group HF+SHT (hydrofluoric acid etching + silane heat treatment): After 90 s of hydrofluoric acid etching, the hybrid blocks were washed and dried, silane was applied and then heated by silane Er:YAG laser irradiation to the parameters in group ER.

Prior to the shear bond strength test, a thin layer of universal adhesive (Clearfil Universal Bond Quick; Kuraray Noritake, Japan) was applied to the specimens (n = 10), and after waiting for 10 s, they were polymerized with a light-emitting diode (LED) light-curing unit (LCU) (1000 mW/cm^2^) (Valo, Ultradent) for 20 s. A nanoceramic resin composite (Zenit, President; Munich, Germany) was filled into a cylindrical teflon mold (2 mm height, 2.4 mm diameter), light cured for 20 s with the LED LCU, and bonded to the hybrid CAD/CAM material. All restorative procedures were performed by a single operator (C.D.) according to the manufacturers’ instructions.

All specimens were thermocycled (SD Mechatronik Thermocycler THE-1100, Mechatronik; Feldkirchen-Westerham, Germany) for 10,000 cycles between 5°C and 55°C (30 s dwell time, 10 s transfer time). They were then subjected to a notch-edge SBS test using a universal testing machine (AGS-X, Shimadzu; Kyoto Japan) with a crosshead speed of 1 mm/min. The load was directly applied on the resin composite/repaired CAD/CAM block interface until fracture occurred. The diameter of the bonded resin composite cylinder was the same as the notched-edge crosshead.^37^ Shear bond strength (SBS) was converted to MPa by dividing the failure load (N) by the bonding area (mm^2^). One operator (Z.O.) who was blinded to the surface treatments used in this study performed all SBS tests.

### Failure Mode Analysis

The failure modes of the specimens were determined using a stereomicroscope (SMZ 1000, Nikon; Tokyo, Japan) under 15X magnification. The mode of failure was determined as “adhesive” if the fracture appeared along the junction of the resin composite and the repaired CAD/CAM block, and “cohesive” if the fracture occurred in the resin composite or repaired CAD/CAM block. Finally, if the fracture appeared along the junction of the resin composite and repaired CAD/CAM block as well as in the composite resin or repaired CAD/CAM block, the mode of failure was determined as “mixed”. One operator (B.O.) who was blinded to the surface treatments used in this study performed all failure mode analyses.

### SEM Analysis

After surface treatments, two specimens from each group were evaluated by SEM. The specimens were gold sputter-coated for an examination of the repaired CAD/CAM block surfaces at an accelerating voltage of 10 kV in secondary mode. The micromorphology of representative surfaces was achieved at 500X and 1000X magnification. One operator (E.E.D.) who was blinded to the surface treatments used in this study performed all SEM procedures.

### Statistical Analysis

Statistical analysis was performed with SPSS 22.0 software. First, the normality of variables was tested by the Shapiro-Wilk test, and the data were then analyzed with the Levene test to determine the homogeneity of variances. Nonparametric tests were used, since they did not satisfy parametric test assumptions. The Kruskal-Wallis test was performed to compare between-group differences according to the surface treatment methods. Similarly, the Mann-Whitney U-test was applied to compare between-group differences according to the presence of silane heat treatment. Statistical significance was set at a confidence level of 0.05 for all analyses.

## Results

The mean SBSs ± SD of all tested groups are presented in Table 2. Regarding the surface treatments, group ER showed significantly lower SBSs than group B and group HF (p < 0.05), while no significant differences in SBSs were determined between groups B and HF (p > 0.05). Furthermore, groups ER+SHT and B+SHT demonstrated significantly lower SBSs than group HF+SHT (p < 0.05), and no significant differences in SBS was observed between groups ER+SHT and B+SHT(p > 0.05). Regarding the silane heat treatment, group B+SHT showed significantly lower SBSs than did group B (p < 0.05). No significant differences were found for the other tested groups (p > 0.05).

**Table 2:** SBS means ± SD and median values (1st-3rd quarter) of all tested groups in MPa (n = 10)

	Treatment						p
	ER		B		HF		
	Mean ± SD	Median	Mean ± SD	Median	Mean ± SD	Median	
Without SHT	6.53 ± 0.61^a^	6.32 [5.98 – 7.18]	12.35 ± 2.24^b^	12.14 [10.21 – 14.4]	9.40 ± 1.18^b^	9.57 [8.85 – 10.13]	<0.001*
With SHT	6.81 ± 0.95^a^	6.81 [5.90 – 7.71]	7.05 ± 1.41^a^	6.67 [5.90 – 8.47]	9.51 ± 2.07^b^	8.33 [8.06 – 11.53]	0.006
p	0.541		<0.001		0.549		

*Different superscript lowercase letters indicate difference within columns. SHT, silane heat treatment; ER: Er:YAG laser treatment; B: bur roughnening; HF: hydrofluoric acid etching.

### Failure Mode Analysis

Table 3 illustrates the failure modes of all groups. The predominant failure mode for most of the groups was adhesive. Group B+SHT exhibited the highest frequency (80%) of adhesive failures, while group HF+SHT exhibited the lowest frequency (10%). Furthermore, group HF+SHT predominantly showed mixed failure mode in repair materials (80%). No cohesive failures were detected for group ER or group ER+SHT. Finally, group B presented cohesive and mixed failure modes in restorative materials at equal rates (20%).

**Table 3:** Failure mode analysis of fractured surfaces (%)

Group	Adhesive (%)	Cohesive (%) (CAD/CAM block)	Cohesive (%) (resin composite)	Mix (%) (CAD/CAM block)	Mix (%) (Resin composite)	Total (%)
ER	60	0	0	0	40	100
ER+SHT	70	0	0	10	20	100
B	60	20	0	20	0	100
B+SHT	80	0	10	0	10	100
HF	60	10	0	0	30	100
HF+SHT	10	10	0	0	80	100

ER: Er:YAG laser treatment; SHT: silane heat treatment; B: bur roughnening; HF: hydrofluoric acid etching.

### SEM Analysis

**Fig 2:**
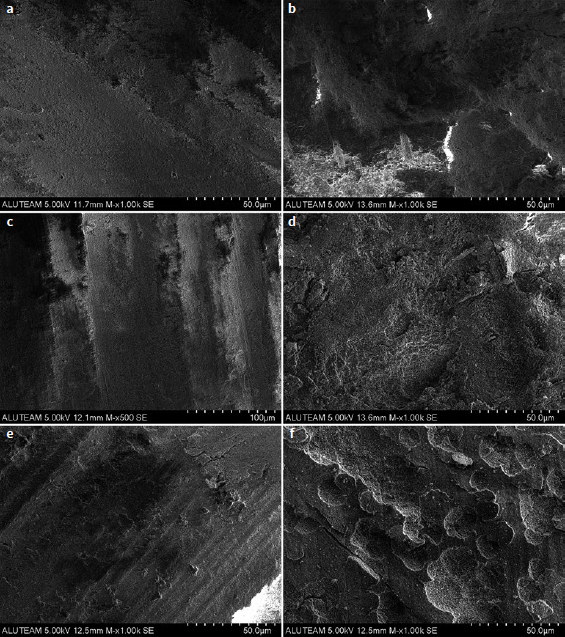
Representative SEM images of all tested groups at 500X and 1000X. a. Group ER; b. Group ER + SHT; c. Group B; d. Group B + SHT; e. Group HF; f. Group HF + SHT. ER: Er:YAG laser treatment; SHT: silane heat treatment; B: bur roughening; HF: hydrofluoric acid etching.

Figure 2 displays representative SEM images of hybrid blocks treated with different surface treatments. Different surface topographies were observed depending on the surface treatment. For example, after bur roughening, parallel scratches and grooves were detected (Fig 2c), while laser-etched groups revealed smoother surface alterations (Fig 2a). Furthermore, surface irregularities were observed for HF-etched groups (Fig 2e), and melted areas were detected with silane heat treatment in the bur-roughened group (Fig 2d).

## Discussion

This study evaluated the effects of different surface treatments and the laser heat treatment of silane on the SBSs of a nanocremic composite to repaired CAD/CAM hybrid blocks. The first null hypothesis, which stated that different surface treatments would not affect the SBS to repaired CAD/CAM hybrid blocks, was rejected because irrespective of silane heat treatment, the Er:YAG laser etching of CAD/CAM blocks led to lower SBSs than did HF etching. The second null hypothesis, which stated that laser heat treatment would not affect the shear bond strength to repaired CAD/CAM hybrid blocks, was rejected because the silane heat treatment negatively affected the SBSs to bur-roughened hybrid blocks, while it did not significantly affect the SBSs to the Er:YAG laser-treated and HF-etched groups.

Repairing the fractured site of dental CAD/CAM restorations is more cost-effective and preserves more remaining dental tissue than replacing a restoration completely.^32^ The repair procedure includes surface pretreatment of the restoration plus silane and adhesive application.^13^ The repair procedures currently used involve chemical and micromechanical bonding, and resin-ceramic adhesion is based on the surface treatment of the ceramic.^2,26^ Surface pretreatments, such as diamond bur abrasion, acid etching^34,44^ and laser etching,^35,42^ have been investigated by simulating the intraoral repair process in the laboratory.^4,23^ In earlier approaches, diamond burs were used to create grooves and undercuts for macromechanical bonding.^11,18^ A silane coupling agent increases the wettability of the ceramic surface and creates effective chemical adhesion between composite resin and ceramic.^6^ Silane has a dual function of bonding with both the methacrylate groups of the resin and the silicon dioxide groups of the ceramic.^28^ Universal dental adhesives have been marketed with a multi-purpose formulation that may adhere to metal, ceramic, and composite restorative materials.^27^ Yoshihara et al^46^ stated that the separate application of silane is more effective than incorporating a silane coupling agent into universal adhesives. Thus, in this study, both a universal adhesive and separate silane application were employed.

Dental resin composite is a popular repair material because of its favorable esthetic and mechanical properties. It has been reported that resin composites adhere well to dental ceramics when the ceramic surface is mechanically treated and a silane coupling agent is applied.^30^ In this study, a nanoceramic resin composite was selected as a repair material, since it has a similar structure to hybrid CAD/CAM materials.

In the oral cavity, chemical degradation, water sorption and the release of residual monomers of the restorative materials may occur over time.^17^ Water sorption and the resulting hydrolysis of the resin might affect adhesive bond strength.^43^ Therefore, following repair procedures, 10,000 thermal cycles (5-55 °C), the equivalent of one year of clinical service,^19^ were conducted to investigate the long-term stability of the bond.

Er:YAG laser application is another approach for conditioning the surfaces of dental restorative materials.^5^ Enhancements in laser technology have enabled the use of laser beams for surface pretreatment through ablation and the melting of ceramic structures, resulting in irregularities on the material’s surface.^10^ Hooshmand et al^26^ demonstrated that applying heat treatments can remove water, alcohol, and other byproducts of the silane coupling agent and help complete the silane condensation reaction. Their results showed that the application of high temperatures to silane-treated ceramic surfaces increased the composite-ceramic bond strength significantly due to the improved alignment of silanol groups. Therefore, they concluded that heat treatment may produce a durable covalent bond. There are different methods for performing the heat treatment of the silane, such as in an oven set at 100°C for 2 min,^33^ hot air application at 50 ± 5°C for 15 s,^16^ or laser application.^9,24^ The parameters of laser application, such as wavelength, frequency, and pulse duration, play a major role.^21^ Sadeghi et al,^38^ who studied the effect of Er:YAG laser surface treatments, and Hakimaneh et al,^24^ who examined the effect of silane heat treatment by Er:YAG laser, both observed that Er:YAG laser application with the parameters 20 Hz, long pulse, 5 W and 250 mJ led to greater surface roughness in feldspathic ceramics than did other parameters. Thus, in this study, these parameters were applied for laser etching the surface as well as silane heat treatment for repairing hybrid CAD/CAM blocks.

In this study, regardless of the presence of silane heat treatments, when comparing the different surface treatments, the Er:YAG laser (5 W/20 Hz, long pulse) resulted in significantly lower SBSs than did hydrofluoric acid (HF). This finding is in line with the increase in cohesive failure rates in HF-treated groups. Furthermore, SEM observations indicated that smoother surface morphologies were obtained with Er:YAG laser-treated groups (Fig 2a). HF etching is the preferred surface pretreatment technique for silica-based ceramic dental restorations.^45^ HF chemically reacts with silica and enhances microretention and wettability. HF etching also reacts with the glass component and exposes hydroxyl groups of the ceramic, which improves their adhesion with monomers.^22,31,44^ The CAD/CAM hybrid material (Cerasmart) used in this study contains both inorganic glass and silica fillers. Additionally, this study found that Er:YAG laser etching caused significantly lower SBSs than did a bur without silane heat treatment. Erdemir et al^14^ reported that 6W laser irradiation produced lower bond strength for repaired lithium-disilicate-reinforced CAD/CAM ceramic material than did a diamond bur. Garshasbzadeh et al^20^ also evaluated the effect of an Er:YAG laser on the surface morphology of an indirect resin composite and reported that applying more than 4W power (20 Hz pulse frequency) might damage the surface due to the high surface temperature produced by the laser. For the bur-roughened group, increased cohesive failure rates are consistent with the SEM images showing parallel scratches found in this study (Fig 2c).

The silane heat treatment with an Er:YAG laser negatively affected the SBSs to bur-roughened hybrid blocks, while there was no significant difference in the laser- and HF-etched groups. Silane heat treatment caused more adhesive failures (80%) in the bur-roughened group. Additionally, SEM observations of melted areas support the failure mode analysis for this group (Fig 2d). Hakimaneh et al^24^ showed that, compared to HF etching and silane treatment, using Er:YAG lasers as a heat source after silanization does not improve the microshear bond strength between composite resin and feldspathic porcelain. In contrast, Hooshmand et al,^26^ Carvalho et al,^7^ and Ergun-Kunt et al^15^ demonstrated that heat treatment significantly increased resin-ceramic bond strength. Hooshmand et al^26^ and Carvolho et al^7^ chose 100°C oven heating as their protocol, while Ergun-Kunt et al^15^ used Er:YAG laser heat treatment on lithium disilicate blocks and measured microtensile bond strength. These different outcomes could be ascribed to methodological differences in heat-treatment procedures and the CAD/CAM block materials used.

## Conclusions

Regarding surface treatments, without silane heat treatment, the Er:YAG laser resulted in significantly lower SBSs than did bur and HF. With silane heat treatment, the Er:YAG and bur presented significantly lower SBSs compared to HF. Regarding the presence of the silane heat treatment, using a bur with silane heat treatment resulted in significantly lower SBSs than did a bur without silane heat treatment.

This study employed an Er:YAG laser at a 20 Hz repetition rate at 5W for 30 s to pretreat surfaces. Thus, further studies should focus on the effect of different pulse frequency, duration and output powers of the Er:YAG laser on the bond strength to repaired hybrid CAD/CAM blocks with the protocol of both silane heat treatment and surface treatment methods.

## Clinical relevance

The silane heat treament had no effect on the repair bond strength to hybrid CAD/CAM materials when hydrofluoric acid and Er:YAG laser were used as surface pretreatment methods. When surfaces are pretreated with bur roughening, silane heat treatment may not be advisable.
